# Assessment of Sleep Quality and Related Factors Using the Pittsburgh Sleep Quality Index (PSQI) Among the General Population in Qassim Region, Saudi Arabia

**DOI:** 10.7759/cureus.113721

**Published:** 2026-07-31

**Authors:** Ashwaq M Almutairi, Noura T Almutairi, Amel A Sulaiman

**Affiliations:** 1 Family Medicine Academy, Qassim Health Cluster, Buraidah, SAU

**Keywords:** caffeine, predictors, psqi, saudi arabia, sleep disturbance, sleep quality, stimulants, young adults

## Abstract

Introduction: Adequate, high-quality sleep is essential for maintaining physical, mental, and emotional health. However, sleep disturbances are increasingly reported among young adults globally. Examining the social, economic, and behavioral determinants of poor sleep is essential to inform targeted public health interventions. This study aimed to assess sleep quality, specific sleep disturbances, and the factors predicting poor sleep patterns among the general population in the Qassim region, Saudi Arabia, using the Pittsburgh Sleep Quality Index (PSQI).

Methods: A descriptive, cross-sectional, community-based study was conducted among 343 adult residents (aged ≥ 18 years) in the Qassim region, Saudi Arabia. Data were collected via an electronically distributed, validated Arabic version of the PSQI questionnaire, which captured sociodemographic characteristics, lifestyle behaviors, and seven distinct domains of sleep. Data analysis was performed using IBM SPSS Statistics for Windows, Version 26.0 (Released 2019; IBM Corp., Armonk, NY, USA).

Results: A total of 343 participants were included in the study. More than half of the participants (58.6%) demonstrated poor sleep quality (PSQI > 5). Sleep latency, disturbance, and daytime dysfunction were the most affected domains. Poor sleep quality was significantly linked to younger age (p = 0.023), student status (p = 0.001), lower income (p = 0.002), and stimulant use (p = 0.006). Multivariate regression analysis indicated that individuals aged >25 years were significantly less likely to report poor sleep (AOR = 0.525; 95% CI: 0.307-0.898; p = 0.019). In contrast, unemployment was associated with a significantly increased likelihood of poor sleep (AOR = 3.271; 95% CI: 1.504-7.114; p = 0.003). Earning 5,000 SAR or more per month lowered the risk (AOR = 0.447; 95% CI: 0.272-0.733; p = 0.001), and using stimulants more than doubled the risk of poor sleep (AOR = 2.272; 95% CI: 1.203-4.181; p = 0.011). Gender, body mass index (BMI), marital status, and chronic disease were not significantly associated with sleep quality (p > 0.05).

Conclusion: Poor sleep quality is common among young adults in Saudi Arabia and is linked to age, employment status, income, and stimulant use. These results indicate the necessity of addressing modifiable behaviors, particularly caffeine consumption, and emphasize the importance of implementing targeted sleep health interventions for younger individuals and those experiencing economic hardship.

## Introduction

Sleep is a fundamental biological process vital for maintaining physical, cognitive, and emotional health, and disturbances in sleep can contribute to profound adverse outcomes. Adequate sleep supports memory consolidation, metabolic regulation, immune function, and emotional stability, while poor sleep quality is associated with neurocognitive impairment, chronic disease, and psychological distress [1-3]. Globally, sleep disturbances affect more than one-third of adults, with higher rates observed in individuals exposed to chronic stress, mental health conditions, and lifestyle disruptions linked to modern living [4,5]. In the Middle East, rapid urbanization, increased screen exposure, and rising rates of obesity and psychological distress have contributed to worsening sleep patterns, including in Saudi Arabia, where significant proportions of students, adults, and healthcare workers report poor sleep quality [6,7]. The Pittsburgh Sleep Quality Index (PSQI) is widely used to assess multidimensional sleep quality and has demonstrated strong reliability and cultural adaptability across populations [8,9].

While several studies in Saudi Arabia have examined sleep quality, they have largely focused on high-stress cohorts, such as medical students and healthcare professionals. There remains a scarcity of community-based studies evaluating multidimensional sleep patterns using the PSQI among the general adult population in the Qassim region. This study aims to address this regional gap.

The objectives of the study were to assess the quality of sleep, disturbances, and factors that affect sleep patterns of the general population in Qassim, Saudi Arabia, using the PSQI, and to determine the estimated prevalence of sleep quality among adults in the Qassim Region using the PSQI global score. Furthermore, the study also describes the pattern of sleep quality components (such as sleep duration, sleep latency, efficiency, disturbances, medication use, and daytime dysfunction) among the study participants and identifies the factors associated with sociodemographic characteristics, lifestyle, behavioral factors, and sleep quality.

## Materials and methods

Study design and setting

This study employed an analytical cross-sectional design to assess sleep quality and identify factors associated with it among the general population in the Qassim region of Saudi Arabia. This design was selected for its effectiveness in capturing data at a specific point in time, making it ideal for analyzing current sleep quality, sleep disturbances, and associated demographic or lifestyle factors. Furthermore, it facilitated the examination of potential associations between demographic factors and sleep quality. The study used the standardized Arabic version of the PSQI to provide a comprehensive evaluation of sleep patterns during the study period. 

Sample size calculation

The sample size was calculated based on a cross-sectional design to estimate the prevalence of poor sleep quality (defined as a PSQI score > 5), using the standard formula for a single proportion:

$n=\frac{Z^{2}\times P\times \left( 1-P \right)}{d^{2}}$

where Z = 1.96 for 95% confidence, P = 0.50 (estimated prevalence for maximum sample size), and d = 0.05 (margin of error). The initial calculation required 384 participants. Adjusting for a 10% non-response or incomplete rate, the final target sample size was rounded to 422 participants. Although the target was 422, 364 surveys were completed. After excluding 21 participants under 18 years of age, the final sample size analyzed was 343 participants. This resulted in an 81.3% response rate.

Sampling technique 

Participants were recruited through convenience sampling using an online platform. The survey was distributed electronically via social media channels (e.g., WhatsApp and Twitter) to reach a broad audience across the Qassim region, including primary healthcare centers (PHCCs), outpatient clinics, workplaces, and public spaces. After providing informed consent, participants completed the questionnaire anonymously. 

Selection criteria 

The study population comprised adults aged 18 years and above who were ambulatory and capable of providing informed consent from the general population of the Qassim region, featuring a mix of Saudi nationals and expatriates; data collection aimed to capture this diversity to ensure a representative sample of the region’s residents. Exclusion criteria included individuals with unmanaged psychiatric disorders, severe chronic diseases that significantly impact sleep, or current use of medications affecting sleep patterns (e.g., sedatives). Additionally, incomplete or invalid questionnaire submissions were excluded to ensure eligibility in the anonymous online environment; mandatory screening questions were placed at the start of the survey. Respondents who indicated they were under 18, had unmanaged psychiatric/severe chronic illness, or used prescribed sleep-modifying medications were automatically directed to an exit screen via Google Forms branching logic.

Data collection 

Data were collected using a structured, self-administered questionnaire consisting of two primary sections: sociodemographic and lifestyle. This included age, gender, nationality, education level, marital status, occupation, monthly income, BMI, smoking status, caffeine intake, physical activity, and chronic illnesses. 

Section 2 included questions from the validated Arabic version of the PSQI, an instrument consisting of 19 self-rated items that assess sleep patterns over the previous month. The items are divided into seven domains: subjective sleep quality, sleep latency, sleep duration, habitual sleep efficiency, sleep disturbances, use of sleep medication, and daytime dysfunction. Each group is scored from 0 to 3, where higher scores mean more severe problems. The scores from all seven domains are summed to obtain a Global PSQI score ranging from 0 to 21. Consistent with the original validation study and subsequent Arabic validation protocols, a global score of 0-5 reflects good sleep quality, whereas a score greater than 5 indicates poor sleep quality [10]. The data were collected from December 2025 to April 2026.

Data analysis 

Statistical analyses were conducted using IBM SPSS Statistics for Windows, Version 26.0 (Released 2019; IBM Corp., Armonk, NY, USA). In this study, the Global PSQI score ranged from 0 to 18. Based on the PSQI cutoff point, those with scores of 0-5 were categorized as “good” sleep quality, while those with scores of 6-18 were categorized as “poor” sleep quality. Descriptive statistics (frequencies, percentages, means, and standard deviations) summarized sociodemographic data and PSQI domain scores. The chi-square test examined the relationship between sleep quality (good or poor) and sociodemographic factors. For inferential and multivariate analyses, age was dichotomized into two categories (≤25 years and >25 years) to optimize statistical power and isolate characteristics unique to the younger adult cohort. A p-value < 0.05 was considered statistically significant. Variables that showed significant associations in this test (age, occupation, monthly income, physical exercise, and stimulant use such as tea and coffee) were included in a multivariate logistic regression to identify independent predictors of poor sleep quality. We reported AORs with 95% CIs to assess the strength of these associations. Variables with p < 0.05 in bivariate screening were entered into the multivariable logistic regression model. Multicollinearity was assessed using variance inflation factors (VIF), with all values resting safely below 1.5. Model fit was confirmed using the Hosmer-Lemeshow test (chi-square = 5.842, p = 0.665), and model discrimination was evaluated using the area under the ROC curve (AUC = 0.712). Missing data resulting from unanswered elective questions (e.g., the physical activity variable, n = 31) were handled using complete-case analysis (listwise exclusion) during inferential modeling, and percentages were calculated based on the total number of valid responses (n = 312) for that specific item.

Ethical considerations 

The study protocol was submitted to the Regional Research Ethics Committee and received ethical approval (letter number: 607/47/008024, registration number: H-04-Q-001). Informed consent was obtained electronically from all participants before they could proceed with the questionnaire. Participation was entirely voluntary, and respondents were informed of their right to withdraw at any time. To ensure confidentiality and anonymity, the data were stored securely in an encrypted database.

## Results

In this study, a total of 343 respondents were eligible for data analysis, resulting in an 81.3% (343/422) response rate (Table 1). Most participants were young, female, and Saudi nationals. The majority of participants were Saudi nationals (95.9%, n = 329), while the remaining 4.1% (n = 14) were non-Saudi expatriates. Nearly 60% were 25 years old or younger, and approximately three-quarters were women. Furthermore, nearly three-quarters were single (73.5%), 78.7% held at least a bachelor's degree, and 53.9% were students. More than half earned less than 5,000 SAR per month. Most participants had a normal BMI (52.2%), although nearly 38% were overweight or obese. Few participants had chronic diseases; 89.5% reported no medical conditions, indicating that the cohort was generally healthy. The reported chronic conditions included hypothyroidism (2.9%), bronchial asthma (1.5%), gastric ulcers (1.2%), anxiety/depression (1.2%), and diabetes/hypertension (0.9%). Additionally, 46.5% engaged in physical activity (31 participants chose not to answer this optional question). Regarding sleep-related habits, only 5.5% reported taking sleep medication, while 85.7% regularly consumed stimulants such as coffee or tea.

**Table 1:** Sociodemographic characteristics of the participants (n = 343)

Study data	N (%)
Age group	
18-20 years	103 (30.0%)
21-25 years	98 (28.6%)
26-30 years	91 (26.5%)
>30 years	51 (14.9%)
Gender	
Male	84 (24.5%)
Female	259 (75.5%)
Nationality	
Saudi	329 (95.9%)
Non-Saudi	14 (4.1%)
Marital status	
Single	252 (73.5%)
Married	76 (22.2%)
Divorced or widowed	15 (4.4%)
Occupation	
Student	185 (53.9%)
Employed	114 (33.2%)
Unemployed	44 (12.8%)
Educational level	
Less than high school	4 (1.2%)
High school	33 (9.6%)
Bachelor’s degree	270 (78.7%)
Postgraduate	36 (10.5%)
Monthly income (SAR)	
<5,000	201 (58.6%)
5,000-10,000	31 (9.0%)
10,001-15,000	26 (7.6%)
>15,000	85 (24.8%)
BMI level	
Underweight (<18.5 kg/m^2^)	34 (9.9%)
Normal (18.5-24.99 kg/m^2^)	179 (52.2%)
Overweight (25-29.9 kg/m^2^)	83 (24.2%)
Obese (≥30 kg/m^2^)	47 (13.7%)
Associated chronic disease	
None	307 (89.5%)
Hypothyroidism	10 (2.9%)
Gastric ulcer	4 (1.2%)
Anxiety and/or depression	4 (1.2%)
Asthma	5 (1.5%)
Diabetes and/or hypertension	3 (0.9%)
Others	10 (2.9%)
Do you perform physical activity? (n = 312)	
Yes	145 (46.5%)
No	167 (53.5%)
Taking medications affecting sleep quality	
Yes	19 (5.5%)
No	324 (94.5%)
Taking stimulants such as coffee or tea that affect sleep quality	
Yes	294 (85.7%)
No	49 (14.3%)

**Figure 1:**
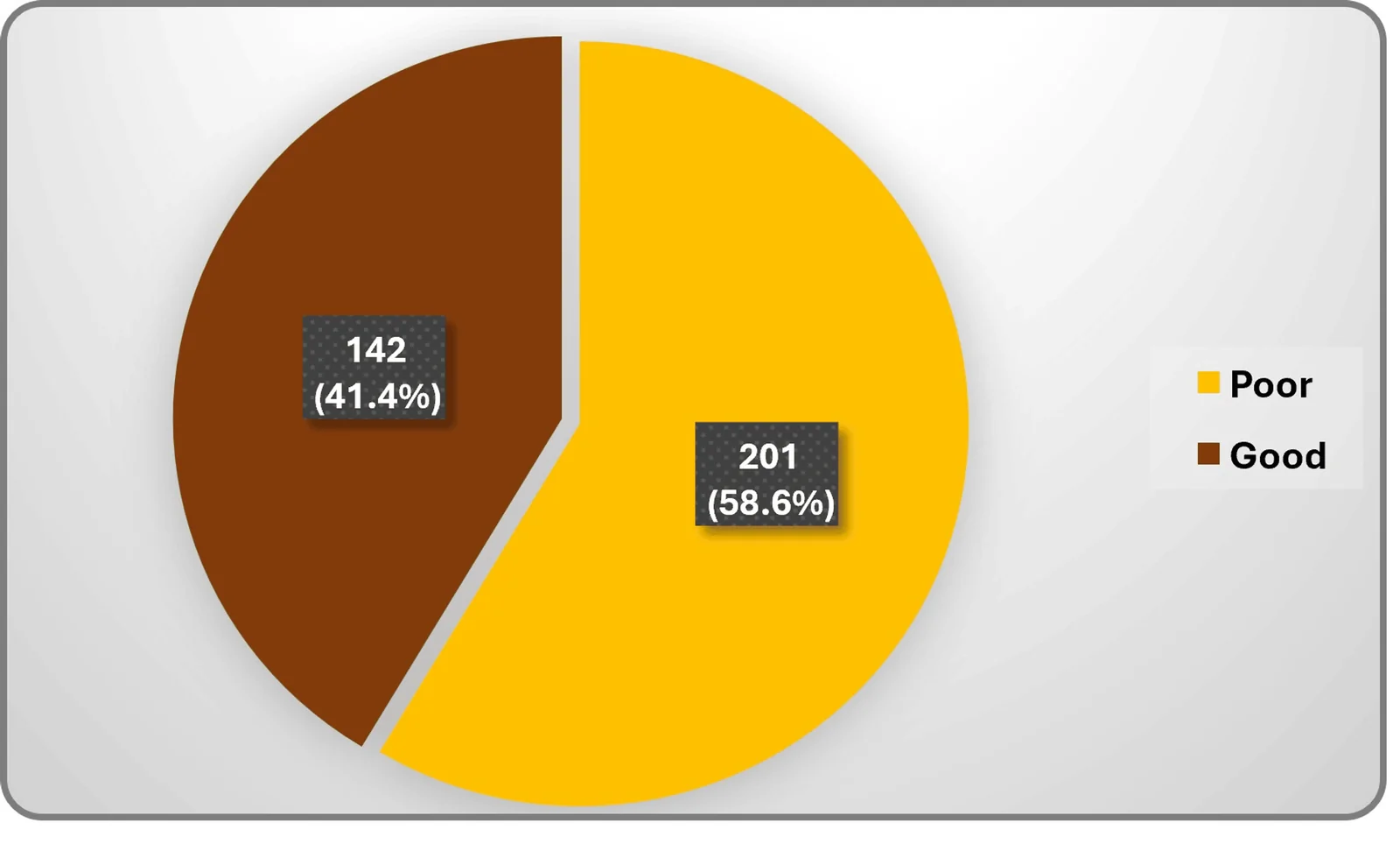
Level of sleep quality based on the assessment using the Pittsburgh Sleep Quality Index (PSQI) (n = 343)

The descriptive statistics of the PSQI among the 343 participants (Table 2) indicate notable variability across sleep domains, with several components reflecting suboptimal sleep patterns. Sleep latency had the highest mean score (1.45 ± 0.97), indicating that many participants experienced difficulty falling asleep. Sleep disturbance (1.25 ± 0.64) and daytime dysfunction (1.08 ± 0.89) also had relatively high mean scores, suggesting that nighttime interruptions and daytime impairment were common. The results further indicate that many participants did not obtain sufficient restorative sleep, as reflected by their sleep efficiency (1.08 ± 1.27) and sleep duration (0.98 ± 0.97) scores. Few participants used sleep medication (0.27 ± 0.68), indicating limited reliance on pharmacological aids. The average subjective sleep quality score was moderate (0.82 ± 0.98). The overall mean global PSQI score was 6.89 ± 3.62, exceeding the standard clinical cutoff of 5 and indicating poor sleep quality in the study population. This finding is consistent with the categorical results, which showed that 58.6% of participants had poor sleep quality, whereas 41.4% had good sleep quality (Figure 1). Overall, these findings suggest that, despite the low use of sleep medication and moderate self-reported sleep quality, factors such as difficulty falling asleep, frequent sleep disturbances, and daytime dysfunction contributed substantially to the high prevalence of poor sleep quality in this cohort.

**Table 2:** Descriptive statistics of the Pittsburgh Sleep Quality Index (PSQI) (n = 343)

Domain	Mean ± SD
Subjective sleep quality	0.82 ± 0.98
Sleep latency	1.45 ± 0.97
Sleep duration	0.98 ± 0.97
Sleep efficiency	1.08 ± 1.27
Sleep disturbance	1.25 ± 0.64
Use of sleep medication	0.27 ± 0.68
Daytime dysfunction	1.08 ± 0.89
Total PSQI score	6.89 ± 3.62

The relationship between sleep quality and sociodemographic characteristics (Table 3) demonstrated several significant associations that help contextualize the sleep patterns observed in this cohort. Age significantly influenced sleep quality, with participants aged 25 years or younger being more likely to report poor sleep (63.7%) than those older than 25 years (36.3%), and this difference was statistically significant (p = 0.023). Occupational status was also significantly associated with sleep quality (p = 0.001), with students comprising the largest proportion of participants with poor sleep (58.7%), whereas employed individuals were more likely to have good sleep quality (44.4%). Monthly income showed a similar pattern. Participants earning less than 5,000 SAR per month had a higher prevalence of poor sleep (65.7%) than those earning 5,000 SAR or more (p = 0.002), suggesting that financial stress may influence sleep patterns. Conversely, engagement in physical activity was positively associated with sleep quality, with 53.1% of physically active participants having good sleep quality compared with 41.8% among those with poor sleep quality (p = 0.048). Stimulant consumption was another significant factor associated with sleep quality (p = 0.006), with 90.0% of participants with poor sleep quality reporting stimulant consumption, compared with 79.6% of those with good sleep quality. Gender, nationality, marital status, education, BMI, chronic disease, and meditation were not significantly associated with sleep quality (all p > 0.05).

**Table 3:** Relationship between the level of sleep quality and the sociodemographic characteristics of the participants (n = 343) p-values were calculated using the chi-square test. **Significant at p < 0.05.

Factor	Category	Poor sleep quality, N (%) (n = 201)	Good sleep quality, N (%) (n = 142)	χ^2^	p-value
Age group	≤25 years	128 (63.7%)	73 (51.4%)	5.167	0.023**
	>25 years	73 (36.3%)	69 (48.6%)		
Gender	Male	45 (22.4%)	39 (27.5%)	1.160	0.282
	Female	156 (77.6%)	103 (72.5%)		
Nationality	Saudi	190 (94.5%)	139 (97.9%)	2.400	0.167
	Non-Saudi	11 (5.5%)	3 (2.1%)		
Marital status	Single	149 (74.1%)	103 (72.5%)	0.108	0.742
	Been married	52 (25.9%)	39 (27.5%)		
Occupational status	Student	118 (58.7%)	67 (47.2%)	14.700	0.001**
	Employed	51 (25.4%)	63 (44.4%)		
	Unemployed	32 (15.9%)	12 (8.5%)		
Educational level	High school or below	18 (9.0%)	19 (13.4%)	1.693	0.193
	Bachelor’s degree or higher	183 (91.0%)	123 (86.6%)		
Monthly income (SAR)	<5,000	132 (65.7%)	69 (48.6%)	10.000	0.002**
	≥5,000	69 (34.3%)	73 (51.4%)		
BMI level	Normal or underweight	126 (62.7%)	87 (61.3%)	0.071	0.790
	Overweight or obese	75 (37.3%)	55 (38.7%)		
Associated chronic disease	Yes	23 (11.4%)	13 (9.2%)	0.464	0.496
	No	178 (88.6%)	129 (90.8%)		
Performed physical activity	Yes	76 (41.8%)	69 (53.1%)	3.905	0.048**
	No	106 (58.2%)	61 (46.9%)		
Medications affecting sleep quality	Yes	14 (7.0%)	5 (3.5%)	1.886	0.170
	No	187 (93.0%)	137 (96.5%)		
Taking stimulants (coffee/tea)	Yes	181 (90.0%)	113 (79.6%)	7.453	0.006**
	No	20 (10.0%)	29 (20.4%)		

The multivariable logistic regression analysis (Table 4) identified several independent predictors of poor sleep quality. Age was a significant predictor of sleep quality, with participants older than 25 years being 47.5% less likely to experience poor sleep than those aged 25 years or younger (AOR = 0.533; 95% CI: 0.307-0.898; p = 0.019). Unemployed participants had more than threefold higher odds of poor sleep than students (AOR = 3.273; 95% CI: 1.504-7.114; p = 0.003), possibly reflecting job instability or related stress.

**Table 4:** Multivariate logistic regression analysis to determine the significant independent predictors of poor sleep quality (n = 343) AOR: adjusted odds ratio. p-values < 0.05 are considered statistically significant.

Factor/independent variable	AOR	95% CI	p-value
Age group			
≤25 years	Ref		
>25 years	0.533	0.307-0.898	0.019
Occupational status			
Student	Ref		
Employed	1.383	0.638-2.993	0.412
Unemployed	3.273	1.504-7.114	0.003
Monthly income (SAR)			
<5,000	Ref		
≥5,000	0.453	0.272-0.733	0.001
Performed physical activity (n = 312)			
Yes	0.653	0.406-1.025	0.064
No	Ref		
Taking stimulants affecting sleep (e.g., coffee/tea)			
Yes	2.273	1.203-4.181	0.011
No	Ref		

Higher monthly income was also associated with a lower likelihood of poor sleep. Participants earning at least 5,000 SAR per month had significantly lower odds of poor sleep (AOR = 0.453; 95% CI: 0.272-0.733; p = 0.001), suggesting that financial stability may be associated with better sleep quality. Consumption of coffee or tea was another significant predictor. Participants who consumed these beverages had more than twice the odds of poor sleep compared with those who did not (AOR = 2.273; 95% CI: 1.203-4.181; p = 0.011), indicating that stimulant consumption may be an important modifiable factor associated with sleep quality. In contrast, being employed was not independently associated with sleep quality compared with being a student (p = 0.412). Furthermore, engagement in physical activity did not remain statistically significant after adjustment in the multivariable regression model (p = 0.064).

## Discussion

The current study provides important insights into sleep quality and its associated factors in a predominantly young, female, and Saudi population. The demographic characteristics of the sample, marked by a high proportion of university-aged individuals, high stimulant consumption, and a low prevalence of chronic illness, are consistent with evidence indicating that younger populations are particularly vulnerable to sleep disturbances arising from academic, psychosocial, and lifestyle-related pressures. Previous research has demonstrated that inadequate sleep adversely affects neurobehavioral functioning, cognitive performance, and emotional regulation in young adults [1-3].

The prevalence of poor sleep quality in our study (58.6%) is comparable to rates reported during periods of heightened stress, such as the COVID-19 pandemic, when sleep disturbances increased across multiple populations [4,5]. Although studies conducted during the peak of the pandemic documented severe sleep disruption driven by acute health-related anxieties, our post-pandemic community-based sample suggests that routine lifestyle factors continue to be significantly associated with sleep quality. Similar prevalence rates have been reported among Saudi medical students [6] and nurses [7]. However, the prevalence observed in our study remains lower than that reported in high-stress clinical populations, including patients with chronic insomnia [11], individuals undergoing methadone maintenance therapy [12], and patients with type 2 diabetes [13]. Unlike these specialized clinical cohorts, our community-based sample provides insight into sleep hygiene among a generally healthy, ambulatory population. Additional studies have reported elevated rates of sleep disturbances among urban Chinese populations [14,15], frontline healthcare professionals [16,17], and patients with orthopedic trauma [18], underscoring the widespread burden of poor sleep. The PSQI domain scores in our study indicated that sleep latency, sleep disturbance, and daytime dysfunction were the most affected components, consistent with findings among young adults and university students worldwide [8,9]. These patterns may reflect irregular sleep schedules, academic demands, evening chronotypes, and stimulant use, all of which have previously been associated with poor sleep quality in university populations [10,19,20]. Similar associations have also been reported among dental students [21], Korean Americans with diabetes [22], and final-year undergraduate students in China [20]. The low use of sleep medication in our sample is consistent with expectations for generally healthy young adults, who typically rely on behavioral rather than pharmacological approaches to managing sleep problems. Although studies from China and Korea have examined sleep quality under different socioeconomic conditions, our findings provide an important baseline for understanding sleep quality in the Qassim region.

Several sociodemographic characteristics were significantly associated with sleep quality. Younger age emerged as a significant predictor of poor sleep quality, consistent with evidence that adolescents and young adults often experience delayed sleep phase tendencies, increased screen time, and greater academic stress [3,9,19]. In contrast, older adults generally exhibit more stable sleep patterns and experience fewer academic stressors. This may explain the lower odds of poor sleep observed among participants older than 25 years in our regression analysis, consistent with findings reported among older adults in Korea [23] and Saudi Arabia [24]. Psychological distress and maladaptive coping strategies have also been shown to adversely affect sleep quality among patients with insomnia [25].

Occupational status was another significant factor, with unemployed individuals demonstrating higher odds of poor sleep quality. This finding is consistent with previous research linking unemployment to psychological distress, financial insecurity, and disrupted daily routines [26,27]. Students also experienced considerable sleep difficulties, supporting evidence from Saudi Arabia and other countries showing that academic pressures, examination-related anxiety, and lifestyle habits negatively affect sleep [6,19,21,28]. Beyond student populations, problematic smartphone use among healthcare workers has likewise been associated with poor sleep quality [29].

Higher monthly income was associated with better sleep quality, consistent with previous studies indicating that socioeconomic stability reduces stress and promotes healthier sleep behaviors [30,31]. Cultural differences in family support systems and financial expectations may partly explain regional variations in this relationship. Sleep disturbances have also been documented among postpartum mothers experiencing depressive symptoms [32], suggesting that emotional distress and caregiving responsibilities may interact with socioeconomic factors to influence sleep quality.

In our study, stimulant consumption was one of the strongest predictors of poor sleep quality. This finding is consistent with evidence showing that caffeine intake prolongs sleep latency, reduces total sleep duration, and decreases sleep efficiency [9]. Studies conducted in Saudi Arabia reinforce this association. Caffeine consumption has been significantly associated with poorer sleep quality among residents of Makkah [33], daily caffeine intake has been negatively associated with sleep quality among medical students [34], and habitual coffee consumption has been linked to poorer sleep quality among Saudi adults [35]. Collectively, these findings identify caffeine consumption as an important modifiable behavioral factor associated with sleep disruption in Saudi populations. Notably, gender, BMI, marital status, and chronic disease status were not significantly associated with sleep quality in our study. This contrasts with previous studies reporting gender-specific differences [27], BMI-related sleep disturbances [21], and impaired sleep among individuals with chronic diseases [10,13]. These discrepancies may reflect the relatively homogeneous and generally healthy characteristics of our sample, the predominantly young age distribution, and the low prevalence of chronic disease.

Finally, the PSQI demonstrated findings consistent with its established validity and reliability across both global and regional populations [36]. Our results are comparable to the strong test-retest reliability reported in contemporary Arabic validation studies [37], which built upon earlier multi-ethnic factor analyses [38] and the foundational Arabic translation developed by Suleiman et al. [39]. These findings further support the robust cultural adaptability of the PSQI for assessing sleep quality among non-clinical populations in Saudi Arabia.

Study limitations 

This study has several limitations. First, convenience sampling through online platforms may have introduced selection bias and limited the generalizability of the findings. Second, the study relied on retrospective self-reported data covering a one-month recall period, making the findings susceptible to both recall and reporting bias. Third, the cross-sectional design captured data at a single time point, precluding causal inference regarding the relationships among stimulant consumption, stress, and sleep quality. Finally, the final sample size was smaller than the calculated target sample, which may have reduced the statistical power to detect smaller effect sizes or subtle differences between subgroups. Future studies involving larger, adequately powered samples are warranted to confirm these findings.

Future research should employ longitudinal designs to monitor changes in sleep quality over time and better elucidate causal pathways among young adults. Additionally, recruiting a more demographically balanced sample, including older adults, male participants, and non-Saudi residents, would improve the generalizability of future findings.

From a public health perspective, comprehensive sleep hygiene interventions should extend beyond recommendations to reduce caffeine consumption. Educational programs should also promote regular physical activity and appropriate timing of caffeine intake. Given the significant association between unemployment and poor sleep quality, healthcare organizations, community centers, and primary care clinics should provide targeted interventions, including sleep health education, stress management programs, and lifestyle counselling for individuals experiencing unemployment or career transitions. Universities and workplaces should likewise implement structured wellness programs tailored to students and young adults, who are particularly vulnerable to sleep deprivation. Finally, integrating routine mental health and sleep assessments into these programs may facilitate early identification of individuals at risk and improve overall well-being.

## Conclusions

This study underscores the high prevalence of poor sleep quality among young adult Saudi females in the Qassim region of Saudi Arabia, identifying unemployment, lower income, and coffee/tea consumption as significant predictors of sleep disruption. While these findings highlight the multifaceted nature of sleep disturbances in this cohort, they also highlight the need for targeted, localized public health interventions and wellness programs. By addressing modifiable behavioral habits alongside socioeconomic vulnerabilities, regional policymakers and educational institutions can develop tailored sleep-health strategies to improve overall sleep quality and health outcomes for similar demographic groups.
